# No association between inactivated influenza vaccination and influenza viral load at diagnosis among young Japanese children: An observational study of the 2013/2014 through 2017/2018 influenza seasons

**DOI:** 10.1111/irv.13213

**Published:** 2023-10-26

**Authors:** Emiko Mukai, Wakaba Fukushima, Saeko Morikawa, Keiko Nakata, Satoshi Hiroi, Masashi Fujioka, Tohru Matsushita, Megumi Kubota, Yoshina Yagi, Tetsuhisa Takechi, Yoshio Takasaki, Shizuo Shindo, Yuji Yamashita, Takato Yokoyama, Yumi Kiyomatsu, Kazuhiro Matsumoto, Akiko Maeda, Kyoko Kondo, Kazuya Ito, Tetsuo Kase, Satoko Ohfuji, Yoshio Hirota

**Affiliations:** ^1^ Department of Public Health Osaka City University Graduate School of Medicine Osaka Japan; ^2^ Department of Public Health Osaka Metropolitan University Graduate School of Medicine Osaka Japan; ^3^ Research Center for Infectious Disease Sciences Osaka Metropolitan University Graduate School of Medicine Osaka Japan; ^4^ Department of Virology Osaka Institute of Public Health Osaka Japan; ^5^ Fujioka Pediatric Clinic Tondabayashi Japan; ^6^ Matsushita Kids' Clinic Kadoma Japan; ^7^ Kubota Children's Clinic Osaka Japan; ^8^ Yagi Pediatric Clinic Yao Japan; ^9^ Takechi Clinic for Pediatrics and Internal Medicine Osaka Japan; ^10^ Takasaki Pediatric Clinic Fukuoka Japan; ^11^ Shindo Children's Clinic Fukuoka Japan; ^12^ Yamashita Pediatric Clinic Itoshima Japan; ^13^ Yokoyama Children's Clinic Kasuga Japan; ^14^ Kiyomatsu Pediatric Clinic Fukuoka Japan; ^15^ Management Bureau Osaka Metropolitan University Hospital Osaka Japan; ^16^ Osaka Metropolitan University Graduate School of Nursing Osaka Japan; ^17^ Clinical Epidemiology Research Center SOUSEIKAI Medical Group (Medical Co. LTA) Fukuoka Japan

**Keywords:** fever, flu vaccine, influenza viral load, young children

## Abstract

**Background:**

The association between inactivated influenza vaccination and viral load in young children remains unclear.

**Methods:**

During the 2013/2014 to 2017/2018 influenza seasons in Japan, children under 6 years of age with pre‐defined influenza‐like illness and influenza‐positive status by real‐time RT‐PCR were recruited at pediatric clinics for this observational study. Influenza viral load was measured for the most predominant subtype/lineage in each season. Using median dichotomized viral load as an outcome, a multilevel logistic regression model was applied to estimate the multivariable adjusted odds ratio (MOR) and 95% confidence interval (CI) for higher viral load.

**Results:**

A total of 1,185 influenza‐positive children were analyzed. The median log_10_ viral load copy number (copies per milliliter) was 5.5 (interquartile range, 4.6 to 6.1) and did not differ by vaccination status: 5.5 for unvaccinated, 5.7 for one dose, and 5.5 for two doses (*p* = 0.67). The MOR of vaccinated (one or two doses) versus unvaccinated children was 1.19 (95% CI: 0.86–1.64). Other factors showing significant associations with higher viral load were positive results for A(H1N1)pdm09 and A(H3N2) in comparison with B/Yamagata. The respective MORs were 3.25 (95% CI: 2.28–4.64) and 1.81 (95% CI: 1.32–2.49). Significantly elevated MORs against higher viral load were also observed for higher body temperature at influenza diagnosis and shorter duration from fever onset to specimen collection.

**Conclusion:**

No association was observed between inactivated‐influenza vaccination and viral load at influenza‐positive diagnosis. Influenza subtype/lineage, body temperature, and time elapsed since fever onset were significantly associated with viral load.

## 1 INTRODUCTION

Influenza still poses a health burden despite the development of medical and pharmaceutical interventions. Annual respiratory deaths associated with influenza virus infection worldwide range from 294,000 to 518,000,^1^ and the most vulnerable populations are children under 59 months of age, older adults, pregnant women, and individuals with underlying diseases.^2^

Influenza vaccination can prevent illness and attenuate the development of severe influenza after breakthrough infections^3^; however, research exploring the association between influenza vaccination and viral load in the upper airway has been limited, with contradictory results. Several studies have reported no association between influenza vaccination and viral load, whereas others have found a significant association, and the results are not generally comparable because of differences across studies in terms of the characteristics of participants and settings.^4^, ^5^, ^6^, ^7^, ^8^

Additionally, even though both young children and older adults are at higher risk of severe influenza,^9^ the clinical manifestations of influenza in these populations differ. Feverishness is observed more frequently among children than older adults,^10^, ^11^ which may be indicative of age‐related differences in viral dynamics. To provide a clearer interpretation, research on the age‐specific association between influenza vaccination and viral load is needed. Our present study explored the factors associated with higher influenza viral load in the upper airway at diagnosis based on the primary endpoint of reduction in viral load by the inactivated influenza vaccine among children under 6 years of age.

## 2 METHODS

### 2.1 Study design and participants

This observational and exploratory study was conducted as a part of a larger ongoing multicenter study monitoring influenza vaccine effectiveness.^12^ A total of 10 pediatric clinics participated from two Japanese prefectures, Osaka and Fukuoka, during five consecutive influenza seasons (2013/2014 to 2017/2018). Participants who met the following criteria were prospectively recruited in a consecutive manner: under 6 years of age and visited a pediatrician with pre‐defined influenza‐like illness (ILI) (defined as temperature >38°C and one of the following respiratory symptoms: cough, runny nose, sore throat, or difficulty breathing) within 7 days of fever onset. The exclusion criteria at recruitment were (1) children under 6 months of age as of September 1 in each season, (2) a history of anaphylaxis after influenza vaccination, (3) previous prescription for antiviral medication against the current ILI, (4) current ILI developed during hospitalization, (5) institutionalization, or (6) living in prefectures other than Osaka or Fukuoka. Among the eligible participants, those with real‐time RT‐PCR‐confirmed influenza were enrolled.

### 2.2 Information collection

All information collection procedures were standardized across the clinics. Information on vaccination status for the current influenza season was derived from the maternal and child health (MCH) handbook or medical records. The MCH handbook is an established tool for sharing children's health and medical information, including immunization status, among family members and medical staff. Almost all families in Japan who have children possess an MCH handbook, which is available in over 50 countries.^13^

Participants in the present study were considered vaccinated if they received an influenza vaccine >14 days prior to ILI onset and considered unvaccinated otherwise. Currently, only an egg‐based inactivated influenza vaccine is approved in Japan. Pediatricians measured each participant's body temperature (°C) at clinic visit. Self‐administered questionnaires answered by parents or legal guardians assessed the following information: prior season's influenza vaccination status, age, sex, history of visiting medical institutions with underlying disease over the preceding 12 months, history of hospitalization over the preceding 12 months, number of ILI symptoms, days after fever onset, and clinics visited. Before participation in the study, we obtained written consent from each child's parents or guardians. All personal information, including individual viral load as described below, was anonymized under the responsibility of the principal investigator (W.F.) in accordance with the Ethical Guidelines for Medical and Health Research Involving Human Subjects.^14^

### 2.3 Copy number for the most predominant influenza virus subtype/lineage

Nasal specimens obtained from the eligible participants by pediatricians using an 8 fr JMS catheter were stored at −20°C until the present RT‐PCR test was carried out at the Osaka Institute of Public Health. After thawing at room temperature, nucleic acids were extracted from 200 μl specimens using the Magtration System with a MagDEA viral DNA/RNA 200 kit (Precision System Science Co., Ltd., Chiba, Japan) and eluted in 50 μl. To distinguish influenza virus subtypes/lineages^15^, ^16^ and quantify the viral load, we performed a one‐step real‐time RT‐PCR method using a Step One PlusTM instrument (Applied Biosystems, Massachusetts, United States). The quantitativity of each real‐time PCR method was evaluated by detecting serial dilutions of quantitated plasmids that contained each target DNA clone. As the target of quantification for viral load, we chose the most predominant virus subtype/lineage in each season, with reference to infectious agents' surveillance reports.^17^, ^18^, ^19^, ^20^, ^21^ In determining the final participants, we excluded individuals who were co‐infected with the most predominant virus subtype/lineage since they could have unrepresentative virus copy numbers.

### 2.4 Statistical analyses

General descriptive statistical methods were used to analyze differences in participant characteristics. Numerical variables are presented as the median and interquartile range (IQR). The Wilcoxon rank sum test or Kruskal–Wallis test was used to explore differences in numerical variables. If statistical significance was observed among multiple comparisons, Bonferroni‐corrected *P* values were calculated to determine which associations were significant. To reduce skewness in the viral load distribution, RNA viral copy number data were log‐transformed. Categorical variables were compared using the chi‐square or Fisher's exact test. To explore factors associated with higher viral load, virus log_10_ copy number data were divided into two categories (high and low viral load) based on the median among all subjects. Considering the hierarchical nature of the data, with individuals nested in clinics, we introduced a multi‐level logistic regression model including 10 factors potentially related to viral load: current influenza vaccination status (unvaccinated/vaccinated), prior influenza vaccination status (no/yes), age (≤2/≥3 years), sex, influenza virus subtype/lineage, body temperature at clinic (<38°C/38.0°C to 38.9°C/≥39.0°C), history of visiting the clinic with underlying diseases in the preceding 12 months (no/yes), history of hospitalization in the preceding 12 months (no/yes), total number of ILI symptoms (1/2/3/4), and time since fever onset (≤2/≥3 days). Each test was two‐sided, and *P* < 0.05 was considered statistically significant. Analyses were performed using SAS (Version 9.4) and R (Version 4.1.2) software.

## 3 RESULTS

**FIGURE 1:**
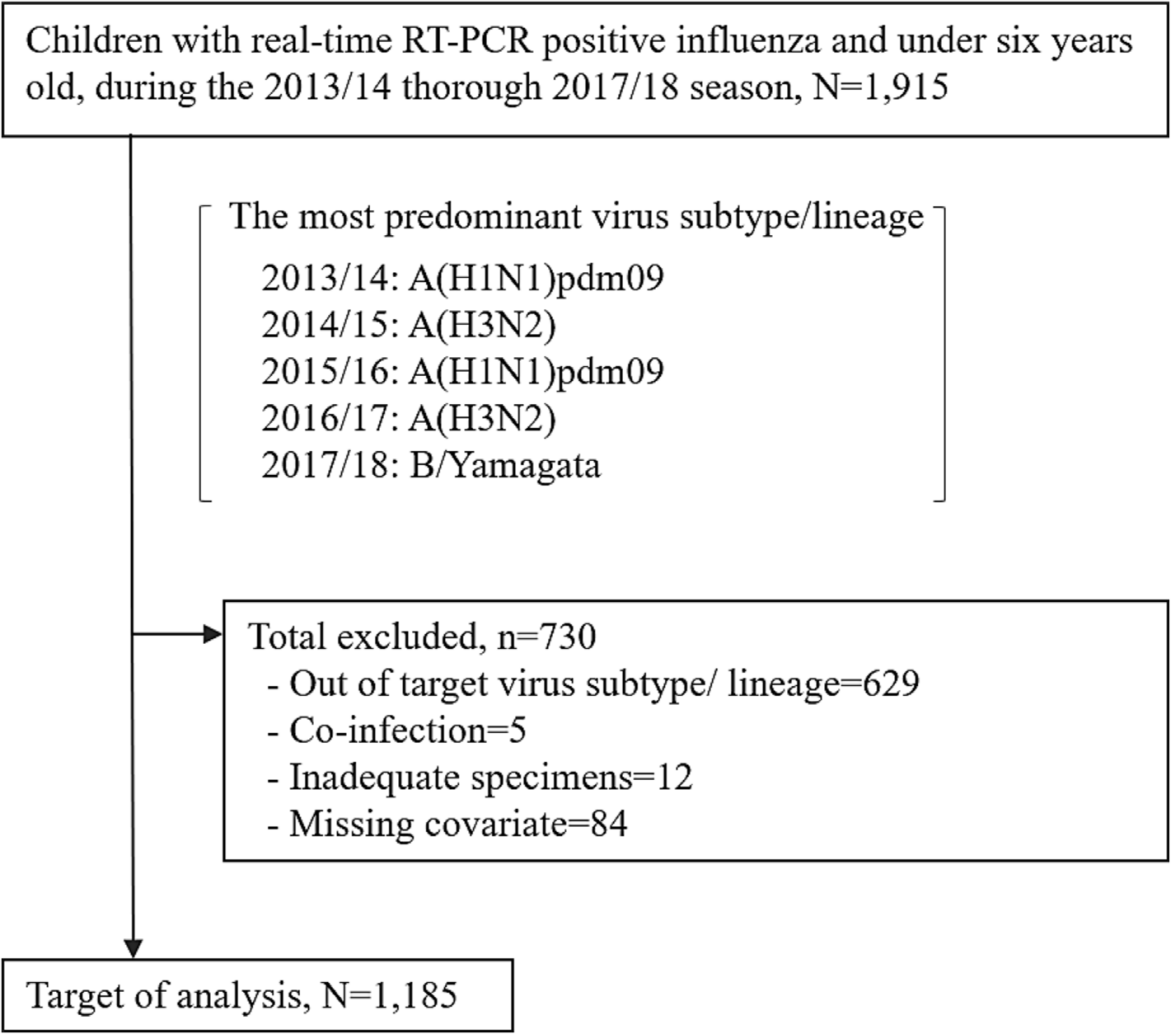
Flowchart of participant enrollment.

We identified 1915 RT‐PCR‐confirmed influenza‐positive children (Figure 1). The most predominant virus subtype/lineage was determined for each season as a target in our analysis: A(H1N1)pdm09 for 2013/2014 and 2015/2016, A(H3N2) for 2014/2015 and 2016/2017, and B/Yamagata for 2017/2018. After exclusions (Table S1), a total of 1,185 participants were included for analysis.

The median age of participants was 3 years (IQR: 2, 4), and the proportion of males was slightly higher (52.1%) than that of females (Table 1). Approximately 35% of participants had been vaccinated with at least one dose. The most common influenza virus subtype/lineage was A(H3N2) (48.6%), and body temperature at the clinic ranged from 38.0°C to 38.9°C for approximately half of the participants (43.4%). The frequencies of visiting a medical institution and hospitalization were low (12.4% and 1.9%, respectively). Almost all participants (95.5%) visited a pediatrician within 2 days of fever onset.

**TABLE 1:** Characteristics of influenza‐positive children under 6 years old during five influenza seasons (2013/2014 through 2017/2018).

	Total, *N* = 1,185	
	*n* or median	(%) or [Q1, Q3]
Current season's influenza vaccination status		
Unvaccinated	765	(64.6)
One dose	119	(10.0)
Two doses	301	(25.4)
Prior season's influenza vaccination status^a^		
Unvaccinated	801	(67.6)
Vaccinated	384	(32.4)
Age, years	3	[2, 4]
0	8	(0.7)
1	210	(17.7)
2	229	(19.3)
3	218	(18.4)
4	273	(23.0)
5	247	(20.8)
Sex		
Male	617	(52.1)
Female	568	(47.9)
Subtype/lineage		
A(H1N1)pdm09	346	(29.2)
A(H3N2)	576	(48.6)
B/Yamagata	263	(22.2)
Body temperature at clinic, °C	38.3	[37.7, 38.9]
<38.0	395	(33.3)
38.0–38.9	514	(43.4)
≥39.0	276	(23.3)
History of visiting medical institution with underlying disease in preceding 12 months^b^		
No	1038	(87.6)
Yes	147	(12.4)
History of hospitalization in preceding 12 months		
No	1162	(98.1)
Yes	23	(1.9)
Number of influenza‐like symptoms^c^		
1	272	(23.0)
2	622	(52.5)
3	247	(20.8)
4	44	(3.7)
Time since fever onset, days		
0	257	(21.7)
1	743	(62.7)
2	132	(11.1)
3	31	(2.6)
4	11	(0.9)
5	9	(0.8)
6	1	(0.1)
7	1	(0.1)
Clinic		
A	169	(14.3)
B	154	(13.0)
C	88	(7.4)
D	68	(5.7)
E	43	(3.6)
F	132	(11.1)
G	139	(11.7)
H	118	(10.0)
I	123	(10.4)
J	151	(12.7)

^a^ Vaccination status in the season prior to the target season (i.e., ‘prior influenza vaccination status of 2013/14 season’ refers to status of the 2012/13 season).

^b^ Including respiratory diseases, heart diseases, renal diseases, neurologic diseases, hematologic diseases, allergies, and immunosuppression.

^c^ Total number of symptoms: cough, runny nose, sore throat, or difficulty breathing.

**FIGURE 2:**
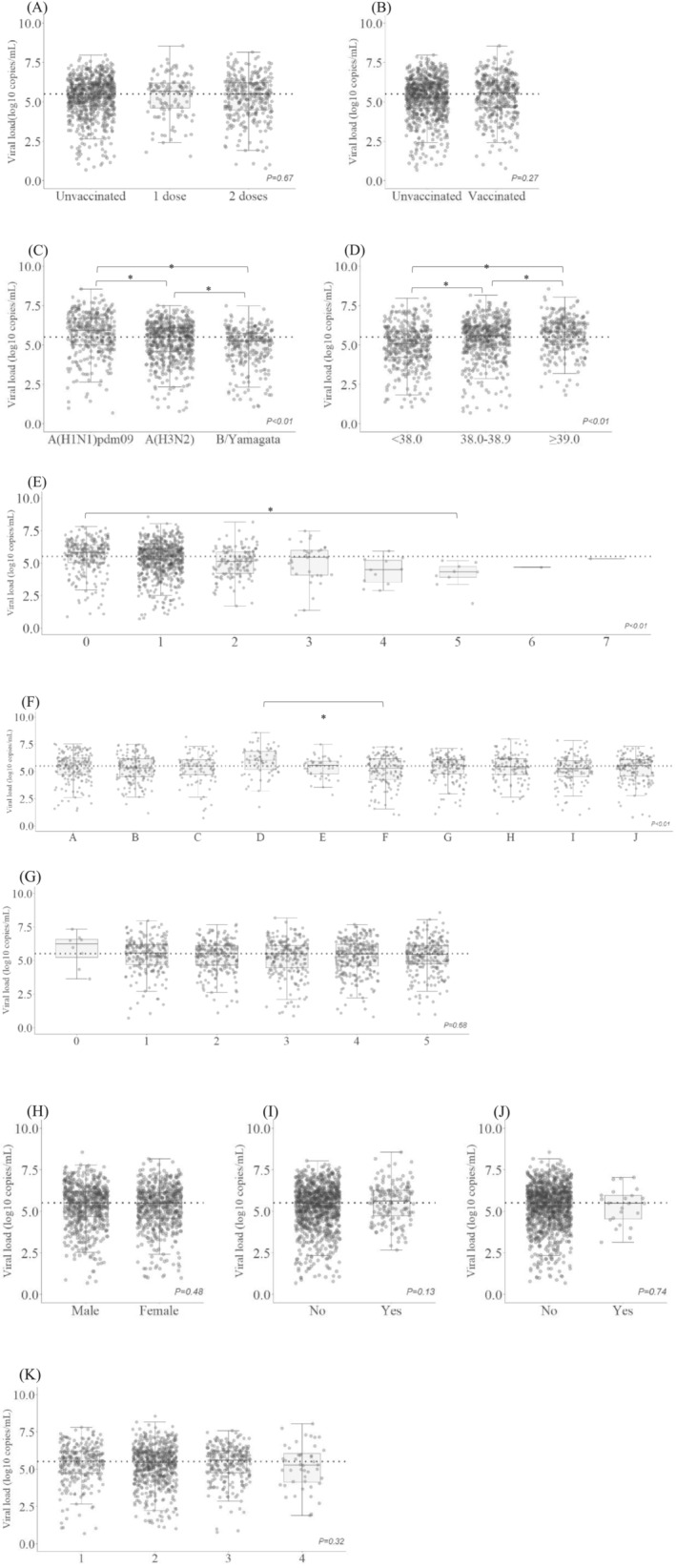
Box‐and‐Whisker plots of viral load according to potentially related factors. (A) Current influenza vaccination. (B) Prior influenza vaccination. (C) Subtype/lineage. (D) Body temperature at clinic, (°C). (E) Time since fever onset (days). (F) Clinic. (G) Age (years). (H) Sex. (I) History of visiting medical institutions with underlying diseases in preceding 12 months. (J) History of hospitalization in preceding 12 months. (K) Total number of influenza‐like symptoms (total number of symptoms: cough, runny nose, nasal congestion, or difficulty breathing). Horizontal bars inside boxes indicate the median, and the lower and upper ends of the boxes are the first and third quartiles. Whiskers indicate values within 1.5× the interquartile range from the upper or lower quartile (or the minimum and maximum if within 1.5× the interquartile range of the quartiles). Gray circles indicate values for individuals. Horizontal dotted lines indicate median viral load for all participants (5.5 log_10_ copies per milliliter). *P* values indicate the results of Wilcoxon rank sum test or Kruskal–Wallis test. A single asterisk indicates statistically significant difference between groups by Bonferroni multiple comparison test.

Among all participants, the median influenza RNA copy number was 5.5 log_10_ copies/mL (IQR: 4.6, 6.1) (Figure 2). The median viral load according to vaccination status for the current season's influenza virus did not significantly differ (5.5 [IQR: 4.7, 6.1] for unvaccinated, 5.7 [IQR: 4.6, 6.2] for one dose, and 5.5 [IQR: 4.5, 6.2] for two doses, *P* = 0.67). Similarly, no significant difference was observed in terms of the distribution based on the prior season's influenza vaccination status. Compared to participants with positive B/Yamagata results, those positive for A(H3N2) and A(H1N1)pdm09 presented with significantly higher median viral loads (5.3 [IQR: 4.3, 5.8], 5.5 [IQR: 4.6, 6.1], and 5.9 [IQR: 5.0, 6.7], respectively; *P* < 0.01). Higher body temperature at the clinic and shorter duration from fever onset were also significantly associated with a higher median viral load. Children with a temperature of >39°C showed the highest median viral load, followed by those with a temperature of 38–39°C or <38°C (5.8 [IQR: 5.0, 6.4], 5.6 [IQR: 4.8, 6.2], and 5.1 [IQR: 4.2, 5.9], respectively; *P* < 0.01). Participants visiting the clinic on the same day of fever onset showed the highest median viral load, which was significantly higher than the load on the fifth day (5.8 [IQR: 5.0, 6.4] and 4.3 [IQR: 3.9, 4.7], respectively; *P* = 0.02). The median viral load of participants who visited clinic D was higher than that of participants who visited any of the other clinics.

The multivariable adjusted model analysis did not find any associations between higher viral load and current influenza vaccination status (Table 2). The multivariable adjusted odds ratio (MOR) of vaccinated children compared with unvaccinated children was 1.19 (95% confidence interval [CI]: 0.86–1.64). Three factors were significantly associated with higher viral load: virus subtype/lineage, body temperature, and time from fever onset to visiting a pediatrician. Positive results for A(H1N1)pdm09 and A(H3N2) presented a significantly higher MOR compared with B/Yamagata positivity: 3.25 (95% CI: 2.28, 4.64) and 1.81 (95% CI: 1.32, 2.49), respectively. Compared with participants with a temperature of <38.0°C, those with a temperature of 38.0–38.9°C or ≥39.0°C showed significantly higher MOR in a temperature‐dependent manner: 2.27 (95% CI: 1.71, 3.01) and 3.01 (95% CI: 2.15, 4.21), respectively (*P* value for trend <0.01). Visiting the clinic within 2 days of fever onset was significantly associated with a higher viral load compared with visiting after 3 days of fever onset.

**TABLE 2:** Distribution of characteristics by dichotomous viral load and factors associated with higher viral load among young children, multilevel logistic regression model.^a^

	Dichotomous viral load,^b^ *N* (%)					Crude OR		Age‐adjusted OR		MOR^c^	
	Low, n = 592		High, n = 593		*P* value^d^	OR	(95% CI)	OR	(95% CI)	OR	(95% CI)
**Current influenza vaccination**											
Unvaccinated	390	(65.9)	375	(63.2)		Reference		Reference		Reference	
Vaccinated	202	(34.1)	218	(36.8)	0.36	1.13	(0.89, 1.43)	1.13	(0.89, 1.44)	1.19	(0.86, 1.64)
**Prior influenza vaccination**											
Unvaccinated	410	(69.3)	391	(65.9)		Reference		Reference		Reference	
Vaccinated	182	(30.7)	202	(34.1)	0.24	1.17	(0.92, 1.50)	1.20	(0.93, 1.54)	1.19	(0.85, 1.66)
**Subtype/lineage**											
A(H1N1)pdm09	133	(22.5)	213	(35.9)		**3.13**	**(2.21, 4.43)**	**3.15**	**(2.22, 4.47)**	**3.25**	**(2.28, 4.64)**
A(H3N2)	288	(48.7)	288	(48.6)		**1.87**	**(1.38, 2.53)**	**1.88**	**(1.39, 2.56)**	**1.81**	**(1.32, 2.49)**
B/Yamagata	171	(28.9)	92	(15.5)	<0.01	Reference		Reference		Reference	
**Body temperature at clinic, °C**											
<38.0	254	(42.9)	141	(23.8)		Reference		Reference		Reference	
38.0–38.9	232	(39.2)	282	(47.6)		**2.22**	**(1.69, 2.92)**	**2.22**	**(1.69, 2.92)**	**2.27**	**(1.71, 3.01)**
≥39.0	106	(17.9)	170	(28.7)	<0.01	**2.94**	**(2.13, 4.07)**	**2.94**	**(2.13, 4.07)**	**3.01**	**(2.15, 4.21)**
						**P for trend <0.01**		**P for trend <0.01**		**P for trend <0.01**	
**Time since fever onset, days**											
≤2	554	(93.6)	578	(97.5)		Reference		Reference		Reference	
≥3	38	(6.4)	15	(2.5)	< 0.01	**0.37**	**(0.20, 0.69)**	**0.37**	**(0.20, 0.68)**	**0.39**	**(0.21, 0.75)**
										(Continued Table 2)	
**Age, years**											
≤2	220	(37.2)	227	(38.3)		Reference		‐		Reference	
≥3	372	(62.8)	366	(61.7)	0.72	0.96	(0.76, 1.22)	‐	‐	0.96	(0.74, 1.24)
**Sex**											
Male	306	(51.7)	311	(52.5)		1.03	(0.82, 1.29)	1.03	(0.82, 1.29)	0.99	(0.78, 1.26)
Female	286	(48.3)	282	(47.6)	0.82	Reference		Reference		Reference	
**History of visiting medical institutions with underlying diseases in preceding 12 months**											
No	523	(88.3)	515	(86.9)		Reference		Reference		Reference	
Yes	69	(11.7)	78	(13.2)	0.48	1.13	(0.79, 1.60)	1.13	(0.80, 1.60)	1.05	(0.71, 1.55)
**History of hospitalization in preceding 12 months**											
No	579	(97.8)	583	(98.3)		Reference		Reference		Reference	
Yes	13	(2.2)	10	(1.7)	0.54	0.74	(0.32, 1.71)	0.74	(0.32, 1.70)	0.62	(0.24, 1.61)
**Number of influenza‐like symptoms**											
1	129	(21.8)	143	(24.1)		Reference		Reference		reference	
2	323	(54.6)	299	(50.4)		0.83	(0.63, 1.11)	0.83	(0.62, 1.11)	0.87	(0.64, 1.18)
3	116	(19.6)	131	(22.1)		1.02	(0.72, 1.45)	1.02	(0.72, 1.45)	1.05	(0.73, 1.52)
4	24	(4.1)	20	(3.4)	0.40	0.76	(0.40, 1.44)	0.76	(0.40, 1.44)	0.80	(0.41, 1.56)
						*P* for trend = 0.39		*P* for trend = 0.38		*P* for trend = 0.57	

*Note*: Statistically significant ORs and 95% CIs are indicated in bold.

Abbreviations: CI, confidence interval; MOR, multivariable adjusted odds ratio; OR, odds ratio.

^a^ Multilevel model analysis was used for individuals (Level 1) nested in clinics (Level 2) to estimate parameters.

^b^ Viral load was divided into two categories by median (low: ≤5.5 log_10_ copies per milliliter and high: >5.5 log_10_ copies per milliliter).

^c^ Model included all variables in the table.

^d^ Chi‐square test or Fisher's exact test.

## 4 DISCUSSION

We examined children <6 years old, a high‐risk group for influenza, to identify factors associated with a higher viral load at influenza‐positive diagnosis based on information obtained from 10 pediatric clinics in two Japanese prefectures over five consecutive influenza seasons. Inactivated influenza vaccination was not associated with viral load, whereas three factors were related to a higher influenza RNA copy number: body temperature at diagnosis of influenza, shorter duration from fever onset, and A(H1N1)pdm09 or A(H3N2) versus B/Yamagata status.

Our finding of no association between inactivated influenza vaccination and influenza viral load in the upper airway is not comparable with previous studies due to study setting differences (e.g., age and health status of participants, timing of specimen collection, and virus subtype/lineage). Several studies sampled specimens only at diagnosis (i.e., one‐time sampling^5^, ^7^, ^8^), as with our study, whereas other studies sampled consecutively at and after diagnosis.^4^, ^6^ Two studies targeted hospitalized patients,^4^, ^8^ whereas ours and three other studies^5^, ^6^, ^7^ targeted outpatients. Regarding virus subtype/lineage, two studies assessed associations of virus subtype/lineage with viral load separately,^4^, ^5^ whereas others simultaneously analyzed associations without distinguishing subtype/lineage, as in our study.^6^, ^7^, ^8^ Despite such setting diversity, to our knowledge, all but one^8^ of these previous studies reported little to no association between influenza vaccination and influenza viral load in the upper airway, consistent with our findings.

The association between specific virus subtype/lineage and higher viral load in this study might be explained by the timing of specimen collection. Previous research exploring the dynamics of influenza viral shedding using consecutively collected specimens indicated that peak viral shedding occurs earlier among patients with influenza A than patients with influenza B, with peak influenza A viral shedding beginning 1 to 2 days after symptom onset.^6^, ^22^, ^23^ In contrast, the highest level of influenza B shedding occurs 3 days after symptom onset or does not exhibit a clear peak.^23^ In our study, >95% of participants visited the clinic within 2 days of fever onset, suggesting that our A(H1N1)pdm09‐ and A(H3N2)‐positive specimens were at the peak of viral shedding, whereas the viral load of B/Yamagata‐positive specimens might have been measured before peak viral shedding. Consequently, virus subtype/lineage‐specific differences in peak viral shedding could explain the significantly higher viral load of A(H1N1)pdm09‐ and A(H3N2)‐positive specimens compared with B/Yamagata specimens.

The positive and monotonic association between influenza viral load and body temperature in this study might be a distinct clinical manifestation among young people, consistent with previous studies. In three studies reporting results similar to ours, the median ages of participants in two studies were 34 (IQR; 13 to 53) years^7^ and 10.1 (range; 0 to 78.8) years,^24^ while the mean age of participants in the third study was 20.0 (standard deviation, ±1.47) years.^25^ In contrast, in a study examining older participants (mean age and standard deviation, 67.08 ± 16.09 years), no positive association was observed.^8^ The biological mechanisms underlying the differences in feverishness across age groups among influenza patients have not been clarified. Cumulative influenza virus infections could lead to protection via cross‐reactive antibodies, which might contribute to a lower likelihood of fever among adults, including older adults.^26^ In contrast, young children with less‐frequent history of influenza might mount a stronger immune response, resulting in a higher likelihood of fever.^27^, ^28^ Our finding of a positive dose–response relationship between viral load and feverishness among children <6 years of age could help elucidate the age‐specific pathophysiology of influenza.

The significant association between higher influenza viral load at diagnosis and shorter duration from fever onset and visiting the clinic in our study was similar to the results of previous studies.^7^, ^29^ Three studies examining the characteristics of influenza viral shedding also supported our present results. A review of 56 studies including 1,280 volunteers in viral challenge experiments reported that viral shedding peaked from 0.5 to 1 day after wild‐type influenza virus inoculation and remained high for 2 days.^22^ Another study of seasonal influenza viral shedding measured serially from index cases and household members showed that viral shedding peaked 1 to 3 days after symptom onset and steadily declined until Days 7 to 9.^6^ A study quantifying influenza RNA copy number and infectious viruses among hospitalized patients reported similar results.^30^

In our current study, we found no association between the total number of ILI symptoms and influenza viral load, which may be explained by the homogeneous health status of participants. Most participants in our study were healthy young children, as >95% and ~90% had no history of hospitalization or visit to a medical institution in the preceding 12 months, respectively. Likewise, a study of healthy university students examining ILI severity scores found no relationship between viral load and ILI severity.^25^ In contrast, two studies reported a significant positive association between viral load and ILI severity among participants with underlying medical issues; in one study, 64% of participants had coexisting medical conditions,^4^ and in the other study, approximately 30% of participants had a higher risk of influenza complications.^7^

Our study has several limitations. We had insufficient information to fully explore the factors associated with higher influenza viral load because data and specimens were initially obtained to assess influenza vaccine effectiveness. Because we limited the quantification to one influenza subtype/lineage for each influenza season, we had to exclude the other subtype/lineage when there were more than two dominant virus subtypes/lineages, which may have affected our results. Our findings cannot be extrapolated to other age groups, including older adults, because our study was restricted to young children. Also, recall bias could have occurred, as most of the information was derived from self‐administrated questionnaires. However, we minimized the possibility of misclassification regarding current influenza vaccination status by obtaining this information from reliable data sources, which would contribute to precise estimations.

In conclusion, we found no association between inactivated‐influenza vaccination and influenza viral load among children under 6 years of age. Further analyses of patients with varying health status and influenza subtype/lineage that reflect the real world could help identify factors associated with a higher influenza viral load.

## AUTHOR CONTRIBUTIONS

**Emiko Mukai:** Data curation; formal analysis; investigation; methodology; writing—original draft; writing—review and editing. **Wakaba Fukushima:** Conceptualization; data curation; funding acquisition; investigation; methodology; supervision; writing—original draft; writing—review and editing. **Saeko Morikawa:** Data curation; investigation; methodology; writing—review and editing. **Keiko Nakata:** Data curation; investigation; methodology; writing—review and editing. **Satoshi Hiroi:** Data curation; investigation; methodology; writing—review and editing. **Masashi Fujioka:** Data curation; investigation; methodology; writing—review and editing. **Tohru Matsushita:** Data curation; investigation; methodology; writing—review and editing. **Megumi Kubota:** Data curation; investigation; methodology; writing—review and editing. **Yoshina Yagi:** Data curation; investigation; methodology; writing—review and editing. **Tetsuhisa Takechi:** Data curation; investigation; methodology; writing—review and editing. **Yoshio Takasaki:** Data curation; investigation; methodology; writing—review and editing. **Shizuo Shindo:** Data curation; investigation; methodology; writing—review and editing. **Yuji Yamashita:** Data curation; investigation; methodology; writing—review and editing. **Takato Yokoyama:** Data curation; investigation; methodology; writing—review and editing. **Yumi Kiyomatsu:** Data curation; investigation; methodology; writing—review and editing. **Kazuhiro Matsumoto:** Methodology; writing—review and editing. **Akiko Maeda:** Methodology; writing—review and editing. **Kyoko Kondo:** Formal analysis; writing—review and editing. **Kazuya Ito:** Methodology; writing—review and editing. **Tetsuo Kase:** Conceptualization; methodology; supervision; writing—review and editing. **Satoko Ohfuji:** Methodology; writing—review and editing. **Yoshio Hirota:** Funding acquisition; supervision; writing—review and editing.

## CONFLICT OF INTEREST STATEMENT

Dr. Kubota reports personal fees from MSD K.K.; Meiji Seika Pharma Co., Ltd.; TORII PHARMACEUTICAL CO., LTD.; Daiichi Sankyo Co., Ltd.; Sanofi K.K.; SHIONOGI & CO., LTD.; and EA Pharma Co., Ltd. and grants from BioGaia AB., outside the submitted work. All the other authors have no conflict of interest to declare.

### PEER REVIEW

The peer review history for this article is available at https://www.webofscience.com/api/gateway/wos/peer-review/10.1111/irv.13213.

## ETHICS STATEMENT

The Ethics Committee of Osaka City University approved the study (No. 2689/approved in 2013, No. 2997/2014, No. 3911/2017, and No. 4416/2019). After obtaining written consent from the children's parents or legal guardians, we invited the children to participate in our multicenter research project to monitor influenza vaccine effectiveness. Although the requirement for individual consent was waived for the current observational study, we offered participants the opportunity to withdraw permission or change their preferences by presenting opt‐out information at each clinic and via the website of the Department of Public Health, Osaka City/Metropolitan University Graduate School of Medicine.

## Supporting information


**Appendix S1.** Supporting Information

## Data Availability

The data presented in this study are available on reasonable request to the corresponding author. The data are not publicly available due to ethical reasons.
