# Supplementary material for: No association between inactivated influenza vaccination and influenza viral load at diagnosis among young Japanese children: An observational study of the 2013/2014 through 2017/2018 influenza seasons
Source: Influenza Other Respir Viruses. 2023 Oct 26;17(10):e13213. doi: 10.1111/irv.13213 (PMC10603291; doi:10.1111/irv.13213)
Supplement: Supplementary file 1 — Appendix S1. Supporting Information [file IRV-17-e13213-s001.docx]

***Appendix:***

Supplementary Table. Predominant virus subtype/lineage and breakdown of exclusions according to influenza season

|  | Total | 2013/14 | 2014/15 | 2015/16 | 2016/17 | 2017/18 |
| --- | --- | --- | --- | --- | --- | --- |
| Initial inclusion | 1915 | 386 | 302 | 424 | 369 | 434 |
| Detected virus subtype/lineage^†^ | **-** | **A(H1N1)pdm09**  A(H3N2)  B/Yamagata  B/Victoria | **A(H3N2)**  B/Yamagata | **A(H1N1)pdm09**  B/Victoria  B/Yamagata  A(H3N2) | **A(H3N2)**  B/Victoria  B/Yamagata  A(H1N1)pdm09 | **B/Yamagata**  A(H3N2)  A(H1N1)pdm09  B/Victoria |
| Predominant virus subtype/lineage for this study | **-** | A(H1N1)pdm09 | A(H3N2) | A(H1N1)pdm09 | A(H3N2) | B/Yamagata |
| Total excluded | 730 | 229 | 23 | 235 | 72 | 171 |
| Out of target  subtype/lineage | 629 | 202 | 4 | 219 | 48 | 156 |
| Co-infection | 5 | 3 | 0 | 2 | 0 | 0 |
| Inadequate specimens | 12 | 6 | 0 | 6 | 0 | 0 |
| Missing covariate | 84 | 18 | 19 | 8 | 24 | 15 |

^†^Bold indicates the predominant subtype/lineage as defined by Japanese Governmental officials (National Institute of Infectious Diseases).
